# Enhanced recovery after radical cystectomy for bladder cancer: a prospective observational case -control study

**DOI:** 10.1186/s12894-026-02093-6

**Published:** 2026-02-25

**Authors:** Marit Habicher, Christina Swoboda, Arne Hauptmann, Christian Koch, Thorsten Diemer, Ann-Catrin Paul, Melanie Markmann, Emmanuel Schneck, Florian Wagenlehner, Matthias Wolff, Michael Sander

**Affiliations:** 1 Department of Anaesthesiology, Operative Intensive Care Medicine and Pain Therapy, Justus Liebig University of Giessen, Rudolf-Buchheim-Street 7, 35392 Giessen, Germany; 2 Department for Urology, Paediatric Urology, and Andrology, Justus Liebig University of Giessen, Rudolf-Buchheim Street 7, 35392 Giessen, Germany

**Keywords:** ERAS, Cystectomy, Outcome

## Abstract

**Introduction:**

The perioperative management of patients undergoing major surgical interventions is a dynamic field, continually striving for advancements in quality, reduction of complications, and cost-effectiveness. The implementation of Enhanced Recovery After Surgery (ERAS) pathways has demonstrated remarkable success in various surgical disciplines, particularly in colorectal procedures. This study investigates whether the implementation of an ERAS protocol for radical cystectomy for bladder cancer at our institution results in a reduced complication rate compared to traditional standards.DRKS00035673 (retrospective) on 13th December 2024.

**Methods:**

This prospective observational case-control pilot study was conducted at the University Hospital Giessen, Germany. Following ethics committee approval, 30 patients were enrolled: 15 patients received perioperative management based on prevailing standard operating procedures, and the subsequent 15 patients were managed according to the newly developed ERAS protocol. The multidisciplinary team included urologists, anaesthesiologists, nursing staff, ICU staff, pharmacists, physiotherapists, dietitians, and administrative staff. Key components of the ERAS protocol included optimized pain management, early nutrition, thrombosis prophylaxis, intraoperative goal directed therapy and early mobilization. The primary outcome was the complication rate until the 30th postoperative day, while secondary outcomes included ICU and hospital mortality, length of stay, and quality of life assessed by the EQ-5D-5L questionnaire.

**Results:**

Thirty patients were included in the final analysis. Patients in the control group underwent radical cystectomy between September 2018 and December 2019, and those in the ERAS group between July 2020 and March 2021. There were no significant differences in basic characteristics between the groups. The primary outcome showed numeric but no statistically significant difference in complication rates (86.7% in the control group vs. 60% in the ERAS group, p=0.21). However, the cumulative POMS score was significantly higher in the control group (7.87 vs. 2.87, p<0.01), and the Clavien-Dindo score was significantly lower in the ERAS group (p=0.02). Quality of life was significantly improved in the ERAS group on the 7th and 30th postoperative day.

**Conclusion:**

Our pilot study suggests feasibility of ERAS implementation in radical cystectomy. The primary endpoint (overall complication rate) did not differ significantly between groups; however, ERAS was associated with lower complication severity and improved early postoperative quality of life, which should be interpreted as exploratory given the small, non-randomised sequential design. Larger multicentre prospective implementation studies are warranted to confirm these findings and to evaluate the influence of protocol adherence and individual ERAS elements.

**Trial registration:**

DRKS00035673 (retrospective) on 13th December 2024

**Supplementary Information:**

The online version contains supplementary material available at 10.1186/s12894-026-02093-6.

## Introduction

Radical cystectomy, a cornerstone in major urological surgery, presents a substantial surgical challenge. The morbidity associated with open radical cystectomy, involving bilateral pelvic lymph node dissection and urinary diversion or bladder reconstruction, reaches significant levels, ranging from 30% to 64% [1] and a 3-month mortality of ~ 3% in historical series, underscoring the need for optimized perioperative pathways [2]. This intricate procedure, characterized by extended surgical times and, on occasion, high volume turnovers, places it among surgeries carrying a high operative risk. Furthermore, the patients undergoing this surgical procedure are older and have more comorbidities than years ago.

Enhanced Recovery After Surgery (ERAS) protocols have been increasingly adapted to RC over the past two decades, and several randomized trials, cohort studies, and meta-analyses now allow RC-specific conclusions.

Two contemporary syntheses provide high-level evidence. In a systematic review and meta-analysis of 22 studies (4048 patients), ERAS use during RC was associated with reduced postoperative morbidity, faster bowel recovery, and a significantly shorter hospital length of stay (LOS). In multivariable models, ERAS shortened LOS by a mean of 4.54 days (95% CI − 5.79 to − 3.28; *p* < 0.001); specific components such as avoidance of nasogastric tubes and use of local anaesthetic blocks were independently linked to shorter LOS [3]. Complementing this, a comprehensive meta-analysis focused on RC found that ERAS protocols reduced LOS (mean difference − 3.46 days; 95% CI − 4.94 to − 1.98), overall complications (OR 0.76; 95% CI 0.61–0.94), and time to first defecation (–1.37 days; 95% CI − 2.06 to − 0.69), without increasing readmissions [4].

Beyond these pooled analyses, RC-specific randomized and prospective studies have shown feasibility and signal of benefit. A randomized pilot trial comparing ERAS to standard care in RC demonstrated improved patient-reported outcomes with similar complication rates, supporting broader ERAS implementation and further trials [5].

A recent meta-analysis evaluated the effectiveness and safety of the ERAS protocol in colorectal surgery. The results showed that ERAS significantly reduced the length of hospital stay by 4.12 days (95% CI: − 5.86 to − 2.38, *p* < 0.00001), postoperative complications (OR = 0.42; 95% CI: 0.27 to 0.65, *p* = 0.0001), and surgical site infections (OR = 0.75; 95% CI: 0.52 to 1.08, *p* < 0.00001) compared to conventional care [6]. The fundamental pillars of this concept encompass modern anaesthesia, optimized surgical techniques, effective pain management, and an intensive postoperative rehabilitation strategy that includes early mobilization and prompt initiation of oral nutrition.

Given this RC-specific evidence base, our study aims to determine whether implementing an ERAS protocol at our institution reduces postoperative complications compared with traditional perioperative care in a contemporary RC cohort.

## Methods

This prospective observational case-control single-centre pilot study was conducted at the University Hospital Giessen, Giessen, Germany (Trial registration: German Clinical Trials Register (DRKS00035673); registration date 13 December 2024; retrospective). Following approval from the local ethics committee of JLU Gießen (reference numbers 135/18 and 73/19), a total of 30 consecutive patients were planned to be enrolled in the study after obtaining written consent.

Inclusion criteria involved written informed consent, age above 18 years, and a planned cystectomy. Exclusion criteria included pregnancy, breastfeeding patients, and those unable to provide consent.

The study comprised two phases. In the initial phase, 15 patients underwent radical cystectomy and received perioperative management based on the prevailing standard operating procedures. Upon completing the first 15 patients, a pre-defined ERAS concept was developed and implemented for the next 15 patients with planned radical cystectomy, who were included and treated according to the new concept.

We implemented an ERAS program for our urology patients by assembling a dedicated multidisciplinary team, including urologists, anaesthesiologists, nursing staff, ICU staff, pharmacists, physiotherapists, dietitians, and administrative staff. Initially, we conducted a comprehensive needs assessment and set clear, measurable goals focusing on reducing hospital stay, minimizing complications, and improving patient satisfaction. We organized thorough training sessions for all team members, to ensure a unified understanding of ERAS protocols. We developed standardized protocols, emphasizing structured perioperative pathway coordination, optimized fluid management, multimodal analgesia, early mobilization, and nutritional support. Notably, no formal structured preoperative patient education program covering individual ERAS components or dedicated preoperative stoma training was implemented (see ERAS section). Regular multidisciplinary meetings were established to discuss patient progress, address challenges, and refine protocols, utilizing digital tools for efficient communication and data sharing.

### Treatment of the control group

The patients in the control group were treated according to the applicable standard operating procedures before implementing the ERAS protocol. This included, among other things, a preoperative routine bowel preparation approximately 36 h before the surgery, leading to an extended period of fasting, as well as fasting postoperatively until evidence of regular bowel function. Additionally, routine premedication with benzodiazepines was part of these SOPs. There were no specific guidelines regarding the anaesthesia procedure. We note that some ERAS-concordant measures (e.g., antibiotic prophylaxis and pharmacologic thromboprophylaxis) were part of standard care, as well as epidural catheter for perioperative pain management in the absence of contraindications. There was no specific protocol for hemodynamic management; instead, it followed the directives of the responsible anaesthesiologist.

### Treatment of the patients with ERAS guidelines

**Fig. 1:**
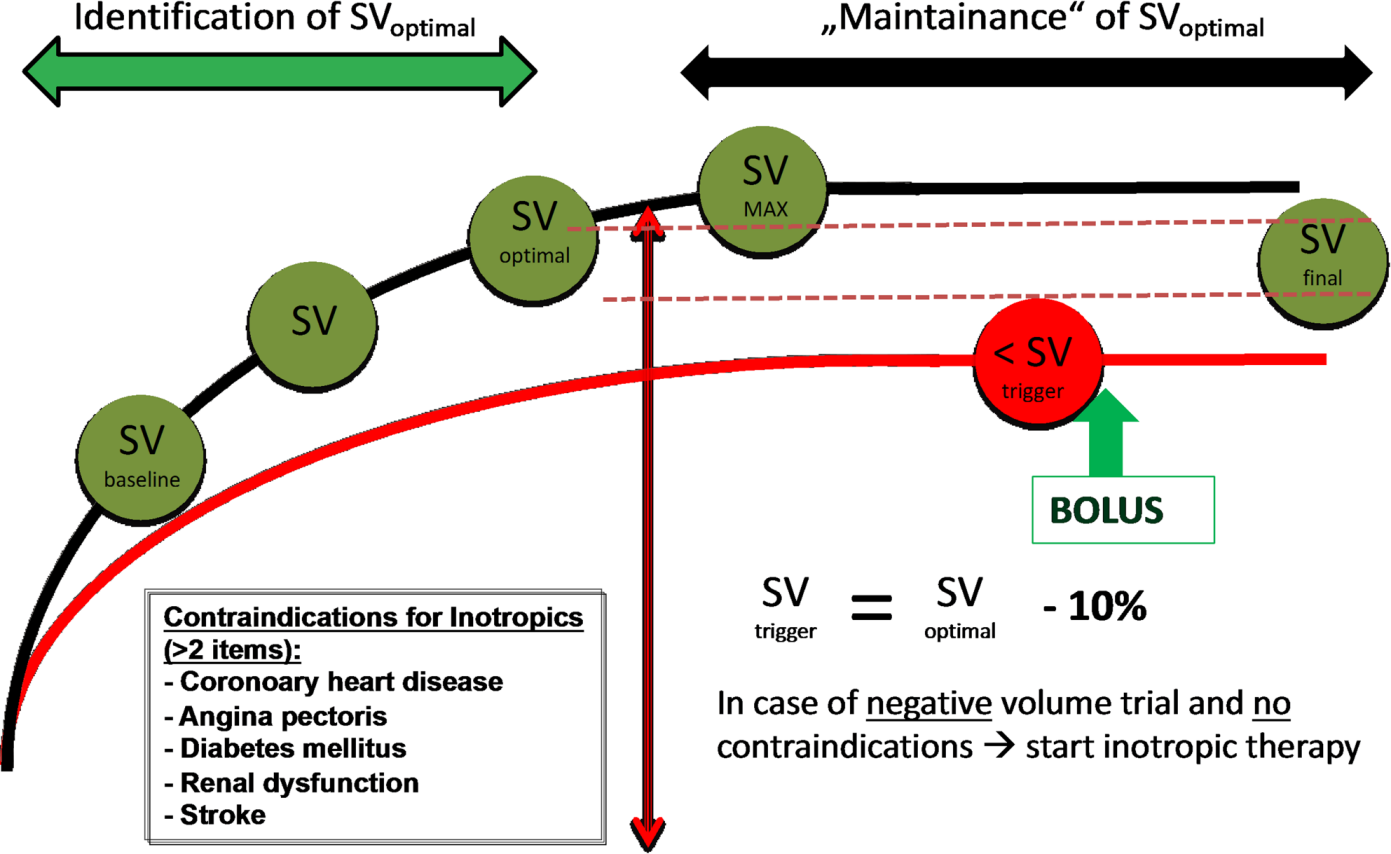
Intraoperative goal directed treatment algorithm of the ERAS Group

The key points of this ERAS concept aim to reduce perioperative stress through the following measures:

1. Optimization of opioid-sparing pain management by the use of an epidural catheter (PDC) and patient-controlled analgesia (PCA).
2. Swift postoperative restoration of bowel function through short fasting periods for solid and liquid food before surgery. Additionally, “carbohydrate loading” with a high-caloric drink is administered both at midnight and 2 hours before the start of the operation. There is no routine preoperative bowel preparation, and postoperative nutrition is initiated as early as the day of surgery, considering regular gum chewing.
3. Thrombosis prophylaxis through early administration of pharmacological thrombosis prophylaxis on the evening before surgery, intermittent pneumatic vein compression during surgery and in the intensive care unit. For patients weighing more than 100 kg, intermittent pneumatic compression is continued 3–4 times per day/night on the peripheral ward until full mobility is achieved. Assisted early mobilization was initiated as early as feasible after surgery (target: evening of surgery or latest postoperative day 1), with daily physiotherapy-supported ambulation goals and minimization of bedrest.
4. No routine premedication with benzodiazepines.
5. Provision of a breathing trainer (spirometer) and targeted instruction for its use by physiotherapists immediately upon admission to the hospital. Daily training for a few minutes throughout the entire hospital stay is recommended.
6. Strict avoidance of perioperative hypo-/hyperthermia.
7. Early perioperative antibiotic prophylaxis (at least 45 min before surgery).
8. Risk-adapted prophylaxis for postoperative nausea and vomiting (PONV).
9. No routine placement of a nasogastric tube.
10. Optimization of fluid therapy through goal directed hemodynamic optimization (Figure 1).
11. Preoperative patient education and stoma care: Preoperative counselling was an integral ERAS element. No formal structured preoperative education program addressing individual ERAS components was conducted, and no dedicated preoperative stoma care training was performed. Stoma care instruction and practical training were routinely provided postoperatively by specialized nursing staff as part of standard ward care and were delivered in both the ERAS and the control group.

The complete ERAS checklist is shown in supplement 1.

### Outcome

- The primary outcome was the composite complication rate until the 30th postoperative day. The recorded complications included MINS (Myocardial Injury after Non-cardiac Surgery), pulmonary complications (pneumonia, pleural effusion, postoperative ventilation), gastrointestinal complications (vomiting, ileus) acute kidney failure, delirium, wound infections, and urological infections (febrile urinary tract infection, bladder tamponade, mucous tamponade, extraluminal) and thrombotic complications (pulmonary embolism or thrombosis).
- Secondary outcome parameters were ICU and hospital mortality, ICU and Hospital length of stay, intraoperative transfusion rate, POMS (Postoperative Morbidity Survey) within 7 days, Health-related Quality of Life evaluated by the EQ-5D-5 L questionnaire, Frailty Index, MMSE (Mini-Mental-Status-Test), Clavien-Dindo Grading System. Surgical procedure and urinary diversion: Urinary diversion type (e.g., ileal conduit, orthotopic neobladder, ureterocutaneostomy) was documented for each patient.

#### Statistics

The primary goal of this pilot trial was to evaluate if the implementation of the ERAS protocol in our clinical routine has potential benefits regarding the outcome of the patients. We conducted a case-control study and compared the pre-existing standard operating procedures for this indication with a newly implemented ERAS protocol, which has been successfully implemented in other patient populations for years. To successfully integrate this protocol at our clinic and demonstrate its clinical benefits, we needed to adopt a study approach that allowed us to present relevant clinical outcomes. A formal sample size calculation was not carried out as this was a pilot project. We planned an analysis after including a total of 30 patients to assess early if changes to our Standard Operating Procedures (SOPs) would show a trend towards better outcomes and if they were clinically feasible.

Descriptive statistics included demographics, clinical characteristics, and hemodynamic data. Normally distributed parameters were presented as mean and standard deviation, while non-normally distributed parameters were described with median and interquartile ranges. Categorical variables were expressed as numbers and percentages. The distribution of parameters was assessed using the Shapiro-test and T-test and Wilcoxon-Test were utilized accordingly for calculating group differences, considering a two-tailed value of *p* < 0.05 to be statistically significant. Given the pilot nature of the study, all secondary outcomes and additional analyses were exploratory; p-values are therefore reported descriptively (two-sided) without adjustment for multiple comparisons. For binary outcomes, absolute risk differences are reported with 95% confidence intervals calculated using the Newcombe method based on Wilson score intervals; for continuous outcomes reported as mean (SD), mean differences are reported with 95% confidence intervals using Welch’s t-method. Categorial variables were tested for statistically significant differences by Fishers exact test. The statistical analysis was carried out using R version 4.3.2 (www.r-project.org).

## Results

In this prospective observational case-control pilot study, we included 30 patients undergoing cystectomy, all of whom were finally analysed. One patient was lost to follow-up after the 30-day follow-up period, resulting in missing data at 6 months and 1 year.

Patients in the control group underwent cystectomy between September 2018 and December 2019, while those in the ERAS group underwent the procedure between July 2020 and March 2021. There were no deaths during the hospital stay. However, within 6 months, 4 patients (28.6%) in the control group and 1 patient (6.7%) in the ERAS group died (*p* = 0.17), whereas after 1 year, 4 patients (28.6%) in the control group and 3 patients (20.0%) in the ERAS group have died (*p* = 0.68).

Compliance with the ERAS protocol during the hospital stay was 78.1%. Detailed representation of compliance and item-level adherence for the ERAS cohort is reported in Supplement 2 to describe implementation fidelity. Compliance in the control period was assessed and compared. Individual ERAS-item adherence was not prospectively captured in the control period; therefore, baseline (pre-implementation) compliance could not be quantified.

Basic characteristics did not differ between the two groups (see Table 1). Intraoperative data are presented in Table 2. Patients in the ERAS group received significantly more colloids (Gelafundin ISO 40 mg/ml, B. Braun) and had a higher intraoperative fluid balance, as they also had lower fluid output. Interestingly, patients in the control group had higher fluid output during surgery and received significantly less norepinephrine.

**Table 1:** Basis characteristics

Age (years)		Control group (n = 15)	ERAS group (n = 15)	*p*
		67.9 ± 10.0	66.8 ± 9.5	0.75
Sex men – n (%)		11 (73.3)	12 (80.0)	1.00
Body height (cm)		171.3 ± 9.4	174.8 ± 6.6	0.25
Body weight (kg)		76.1 ± 13.8	85.1 ± 15.1	0.10
BMI (kg/m^2^)		26.2 ± 5.5	27.6 ± 3.6	0.41
ASA	2	7 (46.7)	6 (40.0)	1.00
	3	8(53.3)	9 (60.0)	
TMN stadium	Tis N0	1 (6.7)	2 (13.3)	0.74
	T1 N0	1 (6.7)	4 (26.7)	
	T2a/b N0/Nx	8 (53.3)	5 (33.3)	
	T3a/b N0/Nx	4 (26.7)	2 (13.3)	
	T4a N0/Nx	1 (6.7)	2 (13.3)	
Degree of Resection (R0/1)	0	12 (80.0)	14 (93.3)	0.60
	1	3 (20.0)	1 (6.7)	
Grading	1	1 (6.7)	0 (0.0)	0.70
	2	4 (26.7)	6 (40.0)	
	3	10 (66.7)	9 (60.0)	
Urinary diversion	Orthotopic ileal neobladder	5 (33.3%)	3 (20.0%)	0.07
	Ileal conduit	2 (13.3%)	8 (53.3%)	
	Ureterocutaneostomy	5 (33.3%)	4 (26.7%)	
	percutaneous nephrostomy	3 (20.0%)	0(0.0)	
Hemoglobin preoperative (g/dL)		13.0 1 ± 1.9	14.1 ± 1.6	0.11
Creatinine preoperative (g/dL)		0.9 [0.8–1.1]	0.9 [0.8–1.2]	0.64
MMSE preoperative		29 [27.5–30.0]	28 [28.0–30.0]	0.98
Frailty	0 (%)	4 (26.7)	6 (40.0)	0.20
	1 (%)	6 (40.0)	6 (40.0)	
	2 (%)	1 (6.7)	3 (20.0)	
	3 (%)	4 (26.7)	0 (0.0)	
EQ-5D-5 L preop Part 1		22 [21.0–23.0]	22 [19.5–23.5]	0.46
EQ-5D-5 L preop Part 2		70 [50.0–85.0]	75 [50.0–80.0]	0.79

Normal distributed values: mean ± standard deviation, non-normal distributed values: median and in square brackets = Interquartile Range, round brackets = Percentage,

Urinary diversion type is also summarized in Table 1. There were no significant differences between the groups.

**Table 2:** Intraoperative data

Anesthesia time (min)	Control group (n = 15)	ERAS group (n = 15)	*p*
	428.7 ± 101.1	457.5 ± 101.3	0.44
Surgery time (min)	303.1 ± 89.1	304.5 ± 54.1	0.96
Fluid input (mL)	4259 [3640.0-4937.0]	4245 [3623.0-5818.5]	0.71
Crystalloids (mL)	3532.1 ± 1121.5	3047.5 ± 1013.0	0.22
*Colloids (mL)*	*500.0 [500.0-1000.0]*	*1400.0 [1000.0-1875.0]*	*< 0.01*
intraoperative Transfusion n (%)	3 (20.0)	3 (20.0)	1.00
Blood loss (mL)	800.0 [575.0-1000.0]	600.0 [400.0-850.0]	0.44
*Overall output (mL)*	*2550.0 [1850.0-3105.0]*	*1000.0 [750.0-1400.0]*	*< 0.01*
*Fluid balance (mL)*	*1805.1 ± 1010.5*	*3577.5 ± 1540.4*	*< 0.01*
cumulative dose of Dobutamine (µg)	0.0 [0.0–0.0]	0.0 [0.0–0.0]	0.16
*cumulative dose of Norepinephrine (µg)*	*1411.2 ± 1053.6*	*3259.9 ± 1559.4*	*< 0.01*

Regarding the primary outcome, overall complications by postoperative day 30 were numerically lower in the ERAS group (9/15, 60.0%) compared with controls (13/15, 86.7%), corresponding to an absolute risk difference of -26.7% points (95% CI -55.9 to + 9.5 Fisher’s exact *p* = 0.21, descriptive). Postoperative pulmonary embolism occurred in 0/15 (0%) ERAS patients versus 3/15 (20.0%) controls (absolute risk difference − 20.0% points, 95% CI -48.6 to + 9.3 Fisher’s exact *p* = 0.22, descriptive). Overall thromboembolic events were 0/15 (0%) in the ERAS group compared with 4/15 (26.7%) in controls (absolute risk difference − 26.7% points, 95% CI -55.2 to + 4.3; Fisher’s exact *p* = 0.10, descriptive). Complications are detailed in Table 3.

**Table 3:** Overall complication rate

overall complications (%)	Control group (n = 15)	ERAS group (n = 15)	*p*
	13 (86.7)	9 (60.0)	0.21
MINS (%)	2 (14.3)	2 (13.3)	1.00
pulmonary complications (%)	3 (20.0)	2 (13.3)	1.00
gastrointestinal complications(%)	7 (46.7)	6 (40.0)	1.00
acute kidney failure (%)	6 (40.0)	3 (20.0)	0.42
delirium (%)	3 (20.0)	2 (13.3)	1.00
wound infections (%)	4 (26.7)	6 (40.0)	0.70
urological infections (%)	6 (40.0)	5 (33.3)	1.00
thromboembolic events (%)	4 (26.7)	0 (0.0)	0.10

In Control group MINS (Myocardial Injury after Non-cardiac Surgery) only 14 patients because of missing data

p-value: differences between the control group and ERAS group

We found a trend toward a longer total hospital stay (*p* = 0.08) in the control group, based on a significantly longer preoperative hospital stay (*p* < 0.01) than those in the ERAS group. However, we did not find differences in postoperative hospital and ICU stay. Furthermore, there was a trend toward more cases with mechanical ventilation needed after surgery in the control group (6 [40%] vs. 1 [6.6%]) (*p* = 0.08).

The cumulative POMS score was higher in the control group (mean 7.87, SD 3.54) than in the ERAS group (mean 2.87, SD 2.80), corresponding to a mean difference of -5.0 points (95% CI -7.39 to -2.61; *p* < 0.01, descriptive). Furthermore, the Clavien-Dindo Grading System scores were significantly lower in the ERAS group (*p* = 0.02). Outcome data are presented in Table 4. In terms of quality of life following surgery, the EQ-5D-5 L questionnaire showed a significant difference on the 7th postoperative day for the first component and on the 30th postoperative day for both components, favouring the ERAS group. Quality of life outcome data are summarized in Table 5.

p-value: differences between the control group and ERAS group.

**Table:** Table 4

Mechanical ventilation after surgery *n* (%)		Control group (n = 15)	ERAS group (n = 15)	*p*
		6 (40.0)	1 (6.7)	0.08
ICU stay (hours)		20 [18–53]	21 [18.5–27.5]	0.88
*preop hospital stay (days)*		*3 [2–3]*	*1 [1–1]*	*< 0.01*
postop hospital stay (days)		26 [18-43.5]	16 [15.5–35]	0.30
overall hospital stay (days)		33 [22.5–47.5]	19 [16.5–36.5]	0.08
*POMS Score*		*7.87 ± 3.54*	*2.87 ± 2.80*	*< 0.01*
MMSE postoperative		29 [25.25-30]	29 [28–30]	0.67
*Clavien-Dindo Grading System*	*Grade I n (%)*	*3 (20)*	*10 (66.7)*	*0.02*
	*Grade II n (%)*	*2 (13.3)*	*0 (0)*	
	*Grade III n (%)*	*4 (26.7)*	*3 (20.0)*	
	*Grade IV n (%)*	*6 (40.0)*	*2 (13.3)*	

*POMS* Postoperative Morbidity Survey,* MMSE *Mini-Mental-Status-Test

**Table:** Table 5

EQ-5D-5 L 7 POD Part 1	Control group (n = 15)	ERAS group (n = 15)	*p*
	16.0 [13.5–17.5]	19.5 [17.3–20.8]	0.03
EQ-5D-5 L 7 POD Part 2	54.7 ± 14.0	56.8 ± 18.8	0.81
*EQ-5D-5 L 30 POD Part 1*	*16.8 ± 3.6*	*20.5 ± 3.6*	*< 0.01*
*EQ-5D-5 L 30 POD Part 2*	*52.0 ± 14.2*	*66.3 ± 13.4*	*< 0.01*
EQ-5D-5 L 6 m Part 1	20.5 [19.0-21.8]	21.0 [18.0–24.0]	0.15
EQ-5D-5 L 6 m Part 2	60.0 [50.0–70.0]	60.0 [60.0–80.0]	0.13
EQ-5D-5 L 12 m Part 1	20.0 [19.0–21.0]	24.0 [19.5–24.0]	0.21
EQ-5D-5 L 12 m Part 2	65.0 [60.0-78.8]	70.0 [70.0-77.5]	0.61

EQ-5D-5 L 6 m Part 1 and Part 2: Data from 7 patients are missing (5 control group, 2 ERAS group), EQ-5D-5 L 12 m Part 1 and Part 2: Data from9 patients are missing (5 control group, 4 ERAS group)

p-value: differences between the control group and ERAS group

## Discussion

In our prospective case-control pilot study, we aimed to investigate whether the implementation of an ERAS protocol in patients undergoing cystectomy could reduce the complication rate compared to a control group receiving traditional care. The results showed a numerical reduction in complications, but no significant difference in the overall complication rate. However, the POMS score, which aggregates various complications, was significantly lower in the ERAS group. Additionally, the Clavien-Dindo score, which classifies severity of surgical complications, was also significantly lower in the ERAS group. This indicates that ERAS pathways in this cohort of patients might prove helpful to reduce the number and severity of postoperative complications.

For secondary outcomes, postoperative quality of life was evaluated using the EQ-5D-5 L questionnaire. The assessment includes two components: first: the descriptive system, which measures five dimensions of health (mobility, self-care, usual activities, pain/discomfort, and anxiety/depression), and the visual analogue scale (VAS), which captures the patient’s overall health perception. A notable improvement was observed in the ERAS group, with a significant enhancement in scores for the descriptive system on postoperative day 7, and in both the descriptive system and VAS on postoperative day 30, indicating a favourable quality of life trajectory in the ERAS cohort.

There are several studies that investigated the implementation of an ERAS protocol for cystectomy and its impact on various outcomes [7–9]. A systematic review and meta-analysis from Feng and colleagues identified 6 studies, including 628 patients, which investigated the clinical efficacy and safety of ERAS in cystectomy patients. They could not show a significant difference in the overall complication rate, however they found significantly difference in recovery of gastrointestinal function after surgery in favour to the ERAS group, as well as a lower incidence in wound infections (*p* = 0.03), and also a trend to a lower incidence in deep vein thrombosis in the ERAS group (*p* = 0.07) [8].

In line with these findings, Ashraf et al. observed that postoperative ileus—one of the most frequent early complications after radical cystectomy—occurred significantly less often in their ERAS cohort (31.9% vs. 55.3%, *p* = 0.021) [9], consistent with prior reports highlighting ileus as a predominant postoperative issue [10]. In our cohort, however, the incidence of gastrointestinal complications, including postoperative ileus, did not differ significantly between ERAS and control groups, which may reflect limited sample size and the uptake of selected ERAS-concordant measures in usual care.

Our study found a significantly lower POMS score in the ERAS group compared to the control group. This significant difference suggests that patients following the ERAS protocol experienced fewer and less severe postoperative complications. Due to the small sample size of this pilot study, we were not able to find statistically significant differences in the overall complication rate, which was only numerically lower in the ERAS group (60%) compared to the control group (86.7%). In line with the study of Feng et al. also in our patient population we found a trend to lower incidence of thromboembolic events (ERAS *n* = 0 vs. Control *n* = 4, *p* = 0.10) and to a lower incidence of pulmonary embolism in the ERAS group [8], however event numbers were very small; therefore this finding should be interpreted cautiously as exploratory rather than evidence of risk reduction.

Another finding from our study which is in line with previous reports was a statistically significantly lower Clavien-Dindo score, which classifies severity of surgical complications, in the ERAS group (*p* = 0.02).

An additional observation was, that the quality-of-life following surgery was superior in the ERAS group, as evaluated by the EQ-5D questionnaire during the early postoperative period. This finding may also be based on the lower complication rate in the ERAS group, contributing to an improved quality of life. However, statistical significance was not achieved in our pilot study, likely attributable to the limited patient population size. A systematic review examining the quality of life and costs associated with ERAS implementation in radical cystectomy patients yielded similar findings regarding the quality of life. The authors, who included four studies in the systematic review, noted a challenge arising from the variability in ERAS protocols and the diverse assessment methods for quality of life. Nevertheless, the authors concluded that the implementation of ERAS was correlated with enhanced patient QoL during the early stages of recovery, particularly in aspects such as the restoration of bowel function, physical/social/cognitive functioning, sleep quality, and pain management [11].

Implementing an ERAS protocol in a urology department, particularly for complex procedures such as radical cystectomy, involves several essential components and professional roles. A multidisciplinary approach is important to ensure comprehensive patient care and effective protocol application. For our urology patients, we established an ERAS program supported by a dedicated team that includes urologists, anaesthesiologists, nursing and ICU staff, pharmacists, physiotherapists, dietitians, and administrative personnel. Adherence to the protocol is a critical factor, as it has been shown to significantly influence patient outcomes. Grilo et al. demonstrated that patients with low protocol compliance (< 65%) had a higher incidence of postoperative ileus (20% vs. 46%, *p* = 0.01) compared to those with high compliance. However, low compliance was not associated with an increased rate of postoperative complications [12]. In our study, we observed a high compliance rate of 78%, which was associated with improved quality of life after surgery and ensured shorter hospital stays. However, we did not observe any differences in gastrointestinal complications, as reported in the study mentioned before.

Protocol adherence is a key determinant of ERAS effectiveness. In our ERAS cohort, item-level compliance was generally high for intraoperative elements, whereas postoperative adherence was more variable, with particularly lower uptake for some recovery-oriented measures (e.g., chewing gum and early diet advancement on the day of surgery, Supplement 2). Incomplete postoperative adherence may have attenuated the potential benefits of ERAS, contributed to within-group variability, and thereby reduced our ability to detect differences in the primary endpoint in this underpowered pilot cohort. These findings underscore the importance of ongoing audit-and-feedback cycles targeting postoperative elements (nutrition and mobilisation) to improve implementation fidelity and to better evaluate the impact of ERAS on clinical outcomes in future studies.

However, intraoperative fluid administration and vasopressor use differed between groups. The ERAS cohort showed greater norepinephrine exposure and a higher net fluid balance; notably, the latter was primarily attributable to lower recorded intraoperative output rather than substantially higher administered volumes. Given the pilot nature of this cohort and the observational design, these differences may reflect variability in measurement and documentation (particularly of output), case mix and surgical complexity, baseline hemodynamic profile, anaesthesiologist preference, or institution-specific management practices, rather than a direct consequence of ERAS implementation. Importantly, the study was not designed to disentangle the effects of individual ERAS elements or to test a specific goal-directed hemodynamic algorithm. Accordingly, any interpretation regarding the impact of goal-directed therapy, vasopressor thresholds, or fluid strategy must remain hypothesis-generating and should be evaluated in adequately powered studies with standardized monitoring, predefined hemodynamic targets, and harmonized output measurement.

We observed no significant differences in mortality rates between the groups; however, given the limited sample size of this pilot study, these findings should be interpreted with caution, as they may lack the power to detect true differences. However, we observed a trend towards a lower mortality within 6 month in the intervention group in comparison with the control group (6.7% vs. 28.6%). In contrast, another observational study assessed the impact of an ERAS pathway on 5-year survival and demonstrated that patients treated according to an ERAS protocol had significantly higher 5-year survival rates than the control group [13].

We may not have observed this significant difference in our study due to our follow-up period only extending to 1 year after surgery.

Another finding was that the pre-operative hospital stay was significantly shorter in the ERAS group, which may be attributed to changes resulting from the ERAS protocol. ERAS patients did not undergo bowel preparation before surgery, whereas the standard group underwent bowel preparation within 48 h before surgery, often under clinical circumstances.

A retrospective analysis of 73 patients undergoing radical cystectomy showed that patients in the ERAS group (*n* = 36) had a significantly shorter hospital stay (7 days vs. 12 days, *p* = 0.003) compared to the other group and experienced better bowel function recovery [14]. Interestingly, we did not observe any significant difference, only a trend, in overall hospital stay in our patients, possibly due to the small sample size. However, our patients had much longer hospital stays compared to those in the previously published study (ERAS 19 days vs. Control 33 days; *p* = 0.08). The significantly longer hospital stays of our patients may also be partially attributed to the German DRG system, which imposes a minimum length of stay. Falling below this minimum results in a deduction in case payment. The so-called average length of stay in Germany for patients undergoing radical cystectomy is approximately 16–18 days after surgery depending on the existing comorbidities. Accordingly, length of stay may be a relatively insensitive endpoint within the German healthcare context, as structural and reimbursement-related factors can substantially influence discharge timing. Patient-centred recovery measures and morbidity severity may therefore provide complementary information beyond LOS.

Furthermore the significantly longer hospital stay of patients in our study cohort can certainly be explained by the fact that in our clinic, patients are only discharged after all urinary catheters and drains have been removed. In other clinics, patients are sometimes discharged with urinary catheters still in place and are then readmitted for their removal.

### Limitations

This study has several limitations that must be acknowledged. The main limitation of this study is the small sample size (*n* = 30), which substantially limits statistical power and limits the generalizability of our findings, particularly for infrequent outcomes such as overall complication rates and mortality. Therefore, the results should be interpreted as exploratory and hypothesis-generating. As a pilot study, it was designed to detect trends rather than provide definitive evidence. In addition, the primary endpoint was a broad composite of heterogeneous complications with different clinical relevance and baseline incidence; in a small pilot cohort this may dilute differential effects and limits interpretability.

The sequential non-randomized design with a historical control group introduces potential selection bias and temporal confounding due to different treatment periods (practice changes, team learning effects) that could influence the results. Urinary diversion types differed numerically between groups and may have acted as a clinically relevant confounder in this sequential, non-randomized comparison. Another possible limitation is that the study was conducted at a single university center, which may limit the applicability of the findings to other institutions with different patient populations, clinical practices, and healthcare systems. Variability in adherence to the ERAS protocol and differences in perioperative care could affect the outcomes. The follow-up period was limited to one year, which may not capture long-term outcomes and complications that could influence the overall assessment of the ERAS protocol’s effectiveness. Extended follow-up periods are necessary to evaluate the durability of the benefits observed in this study. Additionally, our study utilized goal-directed intraoperative therapy focusing on stroke volume optimization. While this approach was intended to improve hemodynamic stability, recent studies, including the IPEGASUS study, have shown that stroke volume optimization does not always lead to improved patient outcomes [15]. This highlights the need for further research to refine intraoperative management strategies within ERAS protocols. Finally, while compliance with the ERAS protocol was relatively high (78.1%), deviations from the protocol were not uncommon and may have influenced the results. Postoperative adherence to ERAS elements in the ERAS group was lower and more variable than intraoperative adherence, which may have attenuated potential ERAS effects and increased within-group heterogeneity. However compliance with individual ERAS elements was not systematically recorded in the historical control period; consequently, we cannot provide a pre-implementation compliance audit or quantify the magnitude of change for each ERAS item over time. Another Limitation is the trial registration was retrospective. No formal structured preoperative patient education on ERAS components or dedicated preoperative stoma training was implemented; stoma education was provided postoperatively in both groups, limiting comparability with ERAS programmes including comprehensive prehabilitation. Future studies should focus on strategies to enhance protocol adherence and assess its impact on outcomes.

## Conclusion

Our study suggests that the implementation of the ERAS protocol in cystectomy patients is feasible in routine clinical practice and may reduce postoperative morbidity and improve quality of life. However, given the small single-centre cohort (*n* = 30) and the non-randomized design, these results should be interpreted as exploratory and hypothesis-generating; the study is underpowered to detect differences in less frequent outcomes such as overall complication rates and mortality, and causal inferences should be avoided. Future work should prioritize adequately powered multicentre prospective implementation studies focusing on adherence, pathway optimization, and patient-centred recovery endpoints; where randomization is feasible, pragmatic designs evaluating implementation strategies may be more appropriate than reverting patients to low-value traditional practices.

## Supplementary Information

Supplementary Material 1.

Supplementary Material 2.

## Data Availability

All data analysed during this study are included in this published article. The datasets used and/or analysed during the current study are available from the corresponding author on reasonable request.

## Abbreviations

BMI: Body mass index
DRG: Diagnosis Related Groups
ERAS: Enhanced Recovery After Surgery
ICU: Intensive care unit
MINS: Myocardial Injury after Non-cardiac Surgery
MMSE: Mini-Mental-Status-Test
PCA: Patient-controlled analgesia
PDC: Epidural catheter
POMS: Postoperative Morbidity Survey
PONV: Postoperative nausea and vomiting
SOPs: Standard Operating Procedures
VAS: Visual analogue scale
