# Supplementary material for: Enhanced recovery after radical cystectomy for bladder cancer: a prospective observational case -control study
Source: BMC Urol. 2026 Feb 25;26:88. doi: 10.1186/s12894-026-02093-6 (PMC13040841; doi:10.1186/s12894-026-02093-6)
Supplement: Supplementary file 1 — Supplementary Material 1. [file 12894_2026_2093_MOESM1_ESM.docx]

***CHECKLIST ERAS for Cystectomy Patients***

**Preoperative on Ward:**

- No preoperative bowel preparation
- Clear carbohydrate drink (ProvideXtra): 10:00 PM the night before and 6:00 AM before surgery
- Thrombosis prophylaxis the night before
- No premedication
- Breathing exercises with spirometer, cough and deep breathing

**Total Preoperative Points: 5**

**Intraoperative:**

- Place Bear Hugger warming blanket under patient before positioning on the OR table
- Administer antibiotics immediately (before induction of anesthesia); repeat after 3 hours of surgery duration
- Epidural catheter
- Evaluate PONV prophylaxis
- Pneumatic venous compression system on the legs
- Prevent hypothermia/hyperthermia (warm cachectic patients during induction)
- Optimized volume therapy according to hemodynamic algorithm ERAS Cystectomy (goal directed therapy)
- Remove nasogastric tube at the end of surgery

**Total Intraoperative Points: 8**

**Postoperative on Day of Surgery:**

- Single dose of antibiotics, in case of bacteriuria for 72 hours
- Chew gum
- Initiate early diet (if possible, order Basic Diet 2)
  - Tea, pudding, soup possible on the evening of surgery
- Aim for early mobilization
  - Sit patient at the edge of the bed in the evening
- Evaluate pain therapy (according WHO pain management guidelines)
- Continue pneumatic venous compression

**Total Postoperative Points (Day of Surgery): 6**

**1st Postoperative Day:**

- For patients >100kg, provide pneumatic venous compression on the ward
  - Use until full mobilization, 3-4 hours daily (daytime/night)
- Evaluate pain therapy (according WHO pain management guidelines)
  - For patients transferred from ICU/IMC: maintain epidural or PCA through ASD
- Physiotherapy
  - Standing exercise for >5 minutes
  - Use of Vibrax device
- Chew gum
- Initiate early diet if possible (Basic Diet 2)
- Use Neostigmine for bowel atony >48 hours: 3 ampoules (15mg) in 500ml Ringer solution

**Total Points from 1st Postoperative Day: 6**
