# Supplementary material for: Enhanced recovery after radical cystectomy for bladder cancer: a prospective observational case -control study
Source: BMC Urol. 2026 Feb 25;26:88. doi: 10.1186/s12894-026-02093-6 (PMC13040841; doi:10.1186/s12894-026-02093-6)
Supplement: Supplementary file 2 — Supplementary Material 2. [file 12894_2026_2093_MOESM2_ESM.docx]

**Supplement 2**

**Overall compliance to the ERAS checklist (ERAS cohort)**

| **Checklist** | **Compliance**  **(mean) %** |
| --- | --- |
| preoperative (max. 5 points) | 73.3 |
| intraoperative (max. 8 points) | 86.7 |
| postoperative (max. 6 points) | 65.6 |
| 1^st^ postoperative day (max. 6 points) | 83.3 |
| overall compliance (max. 25 points) | 78.1 |

**Item-level adherence to the ERAS checklist (ERAS cohort)**

Values are reported as percentage of patients meeting each item (n=15).

| **Checklist** | **Compliance (mean) %** |
| --- | --- |
| **Preoperative on Ward (max. 5 points)** |  |
| No preoperative bowel preparation | 93.3 |
| Clear carbohydrate drink (ProvideXtra): 10:00 PM the night before and 6:00 AM before surgery | 93.3 |
| Thrombosis prophylaxis the night before | 73.3 |
| No premedication | 100.0 |
| Breathing exercises with spirometer, cough and deep breathing | 0.0 |
| **Intraoperative (max. 8 points)** |  |
| Place Bear Hugger warming blanket under patient before positioning on the OR table | 86.7 |
| Administer antibiotics immediately (before induction of anesthesia); repeat after 3 hours of surgery duration | 86.7 |
| Epidural catheter | 66.7 |
| Evaluate PONV prophylaxis | 100.0 |
| Pneumatic venous compression system on the legs | 80.0 |
| Prevent hypothermia/hyperthermia (warm cachectic patients during induction) | 86.7 |
| Optimized volume therapy according to hemodynamic algorithm ERAS Cystectomy (goal directed therapy) | 93.3 |
| Remove nasogastric tube at the end of surgery | 93.3 |
| **Postoperative on Day of Surgery (max. 6 points)** |  |
| Single dose of antibiotics, in case of bacteriuria for 72 hours | 86.7 |
| Chew gum (day of surgery) | 26.7 |
| Initiate early diet (if possible, order Basic Diet 2) (day of surgery) | 40.0 |
| Aim for early mobilization (sit patient at the edge of the bed in the evening) | 80.0 |
| Evaluate pain therapy (according WHO pain management guidelines) | 100.0 |
| Continue pneumatic venous compression | 60.0 |
| **1st Postoperative Day (max. 6 points)** |  |
| Pneumatic venous compression on the ward (1st postoperative day) | 92.9 |
| Evaluate pain therapy (according WHO pain management guidelines) (1st postoperative day) | 85.7 |
| Physiotherapy | 78.6 |
| Chew gum (1st postoperative day) | 42.9 |
| Initiate early diet if possible (Basic Diet 2) (1st postoperative day) | 92.9 |
| Use Neostigmine for bowel atony >48 hours: 3 ampoules (15mg) in 500ml Ringer solution | 100.0 |

Abbreviations: ERAS, Enhanced Recovery After Surgery; PONV, postoperative nausea and vomiting; POD, postoperative day.
